# Impact of disease manifestations on first biologic drug survival in axial spondyloarthritis: a real-life Canadian study

**DOI:** 10.1093/rap/rkaf004

**Published:** 2025-01-08

**Authors:** Raphaël Hurtubise, Sherry Rohekar, Nigil Haroon, Zeynep Baskurt, Tina Chim, Michel Zummer, Robert D Inman, Nicolas Richard, SpondyloArthritis Research Consortium of Canada (SPARCC), Robert D Inman, Dafna Gladman, Nigil Haroon, Vinod Chandran, Sherry Rohekar, Tristan Boyd, Proton Rahman, Nicolas Richard, Michel Zummer, Carter Thorne, Bindu Nair, Shirley Tse, Dianne Mosher, Olga Ziouzina, Alexander Tsoukas, Jonathan Chan, Dax Rumsey, Sibel Aydin, Lihi Eder, Michael Starr, Paul Fortin, Louis Bessette

**Affiliations:** Department of Medicine, Université de Montréal, Montreal, QC, Canada; Division of Rheumatology, Department of Medicine, Western University, London, ON, Canada; Division of Rheumatology, University Health Network, Toronto, ON, Canada; Division of Rheumatology, University Health Network, Toronto, ON, Canada; Division of Rheumatology, University Health Network, Toronto, ON, Canada; Department of Medicine, Université de Montréal, Montreal, QC, Canada; Division of Rheumatology, Hôpital Maisonneuve Rosemont, Montreal, QC, Canada; Division of Rheumatology, University Health Network, Toronto, ON, Canada; Department of Medicine, Université de Montréal, Montreal, QC, Canada; Division of Rheumatology, Hôpital Maisonneuve Rosemont, Montreal, QC, Canada; Centre de Recherche de l’Hôpital Maisonneuve-Rosemont, Montreal, QC, Canada

**Keywords:** ankylosing spondylitis, axial spondyloarthritis, biological therapy, drug survival, anti-TNF

## Abstract

**Objectives:**

The primary objective of this study was to assess the impact of extramusculoskeletal manifestations (EMMs) and peripheral musculoskeletal features on first biologic drug survival in subjects with axial spondyloarthritis (axSpA). The secondary objective was to evaluate the impact of reasons for treatment discontinuation.

**Methods:**

A total of 593 axSpA patients from the SpondyloArthritis Research Consortium of Canada initiating a first biologic drug were identified between 2003 and 2023. Drug survival was presented using Kaplan–Meier curves for each disease manifestation and compared using the logrank test. A Cox proportional hazards model was used to analyse independent predictors of discontinuation. The impact of reasons for treatment discontinuation was assessed using a competing risk analysis.

**Results:**

The presence of psoriasis, nail psoriasis, dactylitis, at least one EMM or at least one peripheral musculoskeletal manifestation was associated with prolonged drug survival compared with subjects without these disease manifestations. In multivariable analysis, psoriasis [hazard ratio (HR) 0.53 (95% CI 0.33, 0.86)] and at least one peripheral musculoskeletal manifestation [HR 0.65 (95% CI 0.47, 0.92)] were independently associated with a lower risk for biologic discontinuation. The presence of psoriasis or dactylitis was associated with reduced treatment discontinuation in patients who stopped their biologic medication for ineffectiveness but not when treatment was discontinued due to adverse events.

**Conclusions:**

This study showed that the presence of some axSpA disease manifestations were associated with prolonged biologic drug survival. Psoriasis and peripheral musculoskeletal manifestations were the most significant predictors of treatment retention. Future research will be needed to further refine treatment strategies according to specific disease manifestations.

Key messages

- Psoriasis and peripheral musculoskeletal manifestations were the most significant predictors of treatment retention in axSpA.
- Psoriasis or dactylitis were associated with reduced treatment discontinuation in patients who stopped for ineffectiveness but not for tolerability.

## Introduction

Axial spondyloarthritis (axSpA) is a chronic inflammatory rheumatic disease affecting the sacroiliac joints and the spine. AxSpA is characterized by various extramusculoskeletal manifestations (EMMs), most commonly acute anterior uveitis (AAU), psoriasis and inflammatory bowel disease (IBD), as well as peripheral musculoskeletal manifestations such as peripheral arthritis, enthesitis and dactylitis [1]. Despite first-line therapy, many patients require the use of advanced treatments, including biologic DMARDs (bDMARDs) such as TNF inhibitors (TNFis) or IL-17 inhibitors (IL-17is) [2].

Biologic drug survival assesses the length of time that patients continue their treatment until discontinuation. It is an important surrogate measure of effectiveness and tolerability in the real-world setting. Previous studies have shown that male sex, younger age, elevated CRP, presence of HLA-B27, radiographic status and being bio-naïve may be factors associated with a better response to treatment and/or drug survival [3–6]. It has also been suggested that peripheral musculoskeletal and EMMs of SpA could influence treatment response, but results are contradictory. The presence of psoriasis, IBD, enthesitis and peripheral arthritis have been associated with worse clinical outcomes [7–11], however, many other studies have not corroborated these findings [4, 6, 9, 12–14]. Few studies have investigated the impact of peripheral musculoskeletal manifestations and EMMs of axSpA on biologic drug survival [15–18] and, overall, the effect of disease manifestations on biologic drug survival remains poorly understood.

The primary objective of this study was to assess the impact of EMMs and peripheral musculoskeletal manifestations on first biologic drug survival. Secondary objectives were to evaluate the impact of reasons for treatment discontinuation on drug survival for each disease manifestation and to compare the biologic drug survival of first TNFis and IL-17is.

## Patients and methods

### Data source

Study subjects were prospectively enrolled in the SpondyloArthritis Research Consortium of Canada (SPARCC) registry. Briefly, they were recruited from 10 sites across Canada and were diagnosed by a rheumatologist experienced in the care of SpA. For the current study, we included all consecutive adult axSpA patients seen between January 2003 and May 2023. Patients were excluded if they had a history of exposure to a bDMARD, if they remained unexposed to a biologic throughout the follow-up period or if they had missing biologic medication start or stop dates.

Approval was obtained from the SPARCC board and local hospital ethics committees for this study. All subjects provided informed written consent to participate in the data collection protocol.

### Exposure and outcome definitions

The exposure of interest was defined as the presence of either AAU, IBD, psoriasis, nail psoriasis, any EMM, peripheral arthritis, enthesitis, dactylitis or any peripheral musculoskeletal manifestation at baseline or at any time point during follow-up as recorded by the study physician (exposed/never exposed). For the period between the last visit prior to the medication stop date, we assumed that exposures remained unchanged, applying the last observation carried forward (LOCF) imputation method. The presence of IBD was defined as a history of Crohn’s disease, ulcerative colitis or prior IBD surgery. Enthesitis was defined as a history of enthesitis or heel pain. Dactylitis was defined as a history of dactylitis, sausage digit or digital tendinitis. Any peripheral musculoskeletal manifestation included peripheral arthritis, enthesitis and/or dactylitis, while any EMM included AAU, IBD, psoriasis or nail psoriasis. Baseline was defined as the initiation date of the first bDMARD.

Drug history was recorded by study physicians at each protocol visit. The outcome of interest for this study was biologic drug survival using reported start and stop dates. Temporary bDMARD interruptions of <90 days were permitted. The main reason for discontinuation was also documented (i.e. lack of efficacy, side effects or other). Patients who switched from a bio-originator to a biosimilar were considered to be on the same biologic drug.

### Clinical covariates

All demographic and disease characteristics, including age, sex, race, smoking history, BMI, depression/anxiety, disease duration, HLA-B27, radiographic status and baseline CRP, were collected at baseline and/or yearly visits according to a standardized data collection protocol. Disease activity at baseline was evaluated using the BASDAI [19] and the Ankylosing Spondylitis Disease Activity Score with CRP (ASDAS-CRP) [20], while functional limitations were assessed with the BASFI [21]. Spinal mobility was measured using the BASMI [22]. Health-related quality of life was assessed with the Ankylosing Spondylitis Quality of Life (ASQoL) score [23] and the 36-item Short Form Health Survey physical and mental component scores (SF-36 PCS and SF-36 MCS, respectively) [24].

### Statistical analysis

Descriptive statistics were used to summarize the baseline characteristics of the cohort. Continuous variables were presented as means with s.d.s and categorical variables were expressed as counts and percentages. The proportion of patients remaining on a biologic drug at selected time points was calculated and drug survival was presented graphically using Kaplan–Meier curves for each exposure of interest. Differences between groups with and without a specific exposure were assessed using the logrank test.

A Cox proportional hazards regression model was used to assess independent predictors of biologic drug survival. Hazard ratios (HRs) were calculated and adjusted for covariates with potential impacts on biologic drug survival and those inferred to be associated with drug survival in the univariable analysis: sex, age, HLA-B27, smoking, depression or anxiety, bDMARD class, psoriasis, nail psoriasis and at least one peripheral musculoskeletal feature. Due to a lack of power, dactylitis was not included in the model and the variable any peripheral musculoskeletal manifestation was chosen since it also included dactylitis. In addition, HLA-B27 and nail psoriasis were incorporated into the model but violated the proportional hazards (PH) assumption, which was tested for each variable using the cox.zph() function. For the covariates violating the PH assumption, stratification was implemented using the strata() function within the Cox model, implemented in the ‘survival’ R package (R Foundation for Statistical Computing, Vienna, Austria) [25]. A stratified Cox model allows the baseline hazard to vary across different levels of the covariate that violated the PH assumption [26], ensuring that the model accounts for the violation. Consequently, HRs for these variables could not be calculated, as their effects were averaged out to ensure the validity of estimates for the remaining variables in the model.

The impact of reasons for treatment discontinuation on drug survival for each disease manifestation was assessed, with a competing risk analysis presenting the probability of event occurrence over time. Cumulative incidence curves were used and comparisons between groups were done using the Gray’s test.

Potentially predictive variables inferred from the univariate survival analysis and clinically relevant variables were included in the multivariable Cox model. In this model, the two variables of interest for our primary objective were psoriasis and any peripheral musculoskeletal manifestation. The other variables were included to control for confounders. The Holm method was performed for multiple testing of two hypotheses using the p.adjust function in the stats R package (R Foundation for Statistical Computing) [27]. The adjusted *P*-values are provided in the results for these variables. All tests were two-sided with a significance level of 0.05. Secondary objective findings should be considered exploratory. Calculations were performed using R Statistical Software (version 4.3.2; R Foundation for Statistical Computing) [28].

## Results

Of the 3393 subjects enrolled across 10 Canadian sites in the SPARCC registry as of 30 May 2023, 2750 individuals were diagnosed with axSpA. Among them, 1318 patients were excluded because they remained unexposed to bDMARDs throughout the follow-up period. Finally, 425 patients were excluded for having been exposed to a bDMARD prior to registry enrolment and an additional 413 patients were further excluded due to insufficient longitudinal data during the follow-up period. The remaining 593 new biologic users with axSpA met the inclusion criteria for this study.

### Demographic and disease characteristics at bDMARD initiation

At bDMARD initiation, the mean age was 38.0 years (s.d. 13.3), 81% were white, 64% were male and the mean disease duration was 15.0 years ( s.d. 11.0) (Table 1). Additionally, 76% were HLA-B27 positive and 87% were classified as radiographic axSpA. A total of 570 subjects started a TNFi and 23 subjects started an IL-17i. The cohort presented the following proportions of disease manifestations at baseline or at any time point during follow-up: AAU, 33%; IBD, 17%; psoriasis, 19%; nail psoriasis, 6%; any EMM, 54%; peripheral arthritis, 17%; enthesitis, 47%; dactylitis, 15%; and any peripheral musculoskeletal feature, 71%.

**Table 1.** Demographic and disease characteristics of the study cohort (*N* = 593).

Variables	Values	Missing data
Age, mean (s.d.), years	38.0 (13.3)	11
Male, *n* (%)	377 (64)	8
White, *n* (%)	470 (81)	15
Ever smoker, *n* (%)	238 (41)	11
BMI, mean (s.d.)	27.5 (6.8)	265
Anxiety and/or depression, *n* (%)	234 (40)	8
Disease duration, mean (s.d.), years	15.0 (11.0)	51
HLA-B27 positive, *n* (%)	390 (76)	81
Radiographic axSpA, *n* (%)	501 (87)	17
CRP, mean (s.d.), mg/l	14.2 (19.5)	307
BASDAI, mean (s.d.)	5.3 (2.2)	307
ASDAS-CRP, mean (s.d.)	3.3 (1.1)	405
BASFI, mean (s.d.)	4.2 (2.6)	310
BASMI, mean (s.d.)	3.0 (1.6)	296
ASQoL, mean (s.d.)	9.0 (5.5)	333
SF-36 PCS, mean (s.d.)	34.9 (10.2)	352
SF-36 MCS, mean (s.d.)	44.7 (11.8)	352
Infliximab, *n* (%)	128 (22)	0
Etanercept, *n* (%)	112 (19)	0
Adalimumab, *n* (%)	220 (37)	0
Golimumab, *n* (%)	97 (16)	0
Certolizumab, *n* (%)	13 (2)	0
Ixekizumab, *n* (%)	1 (0)	0
Secukinumab, *n* (%)	22 (4)	0
csDMARD, *n* (%)	91 (15)	
AAU ever, *n* (%)	196 (33)	1
IBD ever, *n* (%)	101 (17)	0
Psoriasis ever, *n* (%)	110 (19)	1
Nail psoriasis ever, *n* (%)	33 (6)	1
Any EMM ever, *n* (%)	322 (54)	0
Peripheral arthritis ever, *n* (%)	97 (17)	30
Enthesitis ever, *n* (%)	281 (47)	0
Dactylitis ever, *n* (%)	91 (15)	0
Any peripheral musculoskeletal feature ever, *n* (%)	420 (71)	0

csDMARD: conventional synthetic disease-modifying antirheumatic drug.

Age, sex, race, BMI, disease duration, HLA-B27, radiographic classification, CRP, BASDAI, ASDAS-CRP, BASFI, BASMI, ASQoL, SF-36 PCS, SF-36 MCS and medication data were baseline values, while smoking, anxiety and/or depression and disease manifestations were exposed/never-exposed variables.

Compared with their counterparts without the disease manifestation, HLA-B27 positivity was more frequently found in subjects with AAU (85% *vs* 72%), while it was less frequently found in subjects with IBD (68% *vs* 78%) (Supplementary Table S1, available at *Rheumatology Advances in Practice* online). A higher proportion of patients with IBD also exhibited a history of peripheral arthritis (24% *vs* 16%). Subjects with psoriasis presented more nail psoriasis (20% *vs* 2%) and dactylitis (22% *vs* 14%) compared with those without. Furthermore, subjects with nail psoriasis were more often diagnosed with psoriasis (67% *vs* 16%), enthesitis (67% *vs* 46%), dactylitis (30% *vs* 14%) and at least one peripheral musculoskeletal feature (91% *vs* 70%) (Supplementary Table S2, available at *Rheumatology Advances in Practice* online). Subjects with peripheral arthritis had more history of IBD (24% *vs* 16%), enthesitis (57% *vs* 45%) and dactylitis (26% *vs* 13%) compared with those without peripheral arthritis. A history of dactylitis was most often found in subjects with enthesitis (23% *vs* 8%), and a higher proportion of participants with dactylitis had a history of nail psoriasis (11% *vs* 5%), peripheral arthritis (29% *vs* 15%) and enthesitis (73% *vs* 43%) (Supplementary Table S3, available at *Rheumatology Advances in Practice* online). Full disease characteristics according to disease manifestations are provided in Supplementary Tables S1–S3, available at *Rheumatology Advances in Practice* online.

### Biologic drug survival

**Figure 1.**
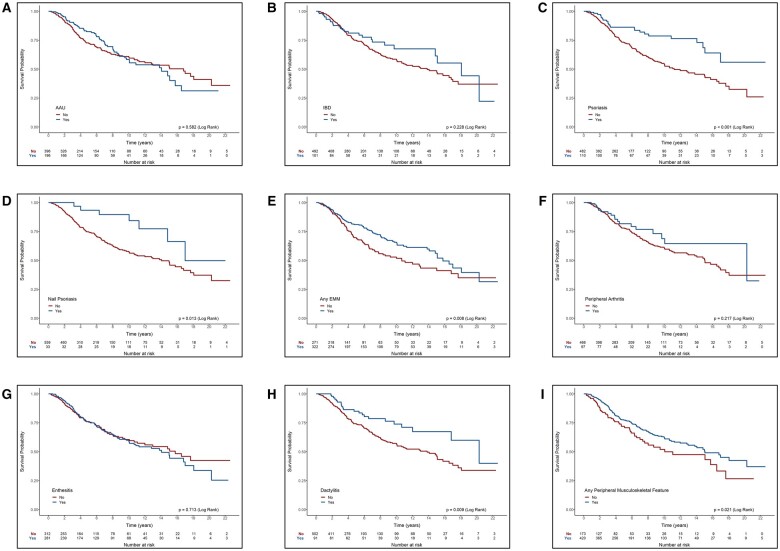
Kaplan–Meier biologic drug survival curves according to **(A)** acute anterior uveitis, **(B)** inflammatory bowel disease, **(C)** psoriasis, **(D)** nail psoriasis, **(E)** any EMM, **(F)** peripheral arthritis, **(G)** enthesitis, **(H)** dactylitis and **(I)** any peripheral musculoskeletal manifestation

Subjects with psoriasis, nail psoriasis, dactylitis, any peripheral musculoskeletal manifestation or any EMM had longer biologic drug survival compared with those without these disease manifestations, with logrank *P*-values of <0.001, 0.013, 0.009, 0.008 and 0.022, respectively. (Fig. 1C, D, G–I). AAU, IBD, peripheral arthritis and enthesitis had no significant effect on biologic drug survival (Fig. 1A, B, E, F).

### Predictors for drug discontinuation

Multivariable analysis showed that psoriasis [HR 0.53 (95% CI 0.33, 0.86)] and at least one peripheral musculoskeletal manifestation [HR 0.65 (95% CI 0.47, 0.92)] were disease characteristics independently associated with a lower risk to discontinue the biologic treatment (Table 2). Of note, the risk of discontinuing treatment was similar between subjects on TNFi or IL-17i.

**Table 2.** HRs for first bDMARD discontinuation using Cox proportional hazards models.

Variables	Univariable, HR (95% CI)	*P*-value	Multivariable, HR^a^ (95% CI)	*P*-value
Sex (male:female)	0.52 (0.39, 0.69)	<0.001	0.43 (0.31, 0.60)	<0.001
Age	1.00 (0.99, 1.01)	0.960	1.01 (0.99, 1.02)	0.35
Psoriasis	0.45 (0.29, 0.69)	<0.001	0.53 (0.33, 0.86)	0.02
Any peripheral musculoskeletal feature	0.70 (0.51, 0.95)	0.022	0.65 (0.47, 0.92)	0.02
bDMARD class (TNFi *vs* IL-17i)	0.66 (0.24, 1.79)	0.41	0.52 (0.19, 1.45)	0.21
Smoking	1.36 (1.02, 1.82)	0.036	1.47 (1.07, 2.03)	0.018
Anxiety and/or depression	0.72 (0.53, 0.99)	0.045	0.63 (0.44, 0.91)	0.015

*P*-values for psoriasis and any peripheral musculoskeletal feature are adjusted for multiplicity using the Holm method. The unadjusted *P*-values were 0.010 and 0.014, respectively.

a Model was adjusted for the following variables: sex, age, HLA-B27, psoriasis, nail psoriasis, any peripheral musculoskeletal feature and the class of bDMARD used.

### Reasons for biologic drug discontinuation

During the study period, a total of 191 patients (32%) stopped their first biologic drug. Of those, 187/570 TNFi users and 4/23 IL-17i users stopped their treatment. Most patients discontinued for lack of effectiveness (*n* = 119) or side effects (*n* = 30), while the remainder stopped their drug for other reasons (*n* = 31) or had unavailable data (*n* = 11). Of those who discontinued due to a lack of effectiveness, 3% did so within 6 months and were categorized as primary non-responders, while 97% were classified as secondary non-responders, having discontinued their treatment after 6 months.

**Figure 2.**
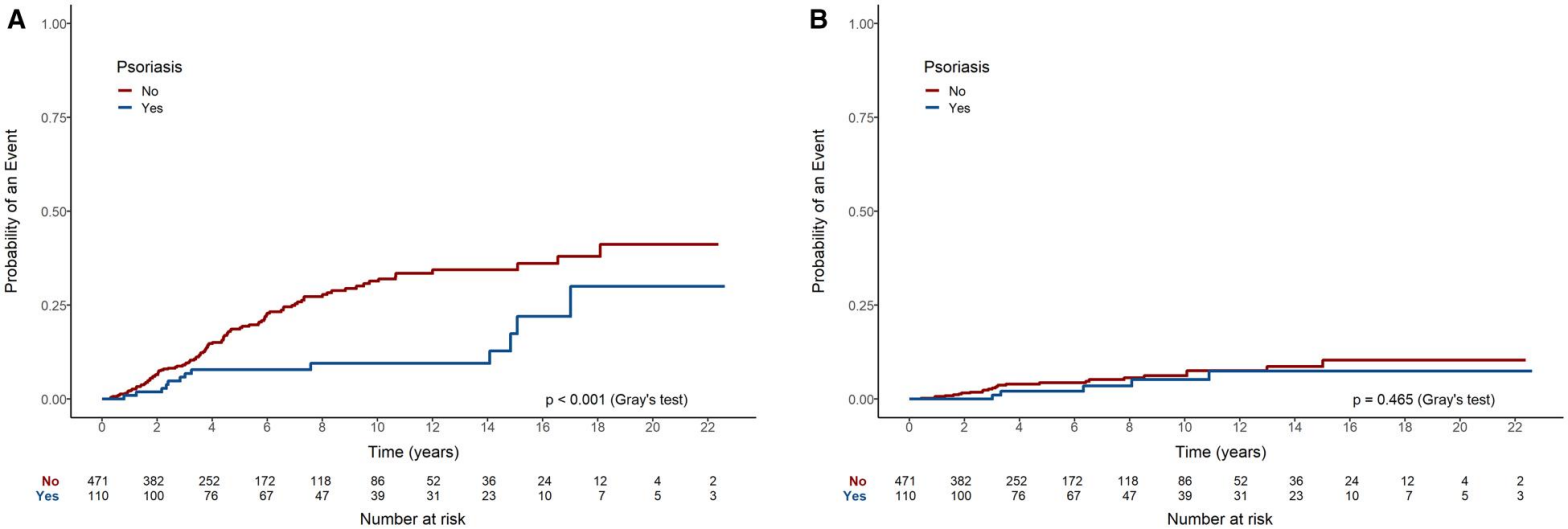
Occurrence of treatment discontinuation over time in patients with psoriasis due to **(A)** lack of effectiveness and **(B)** side effects

**Figure 3.**
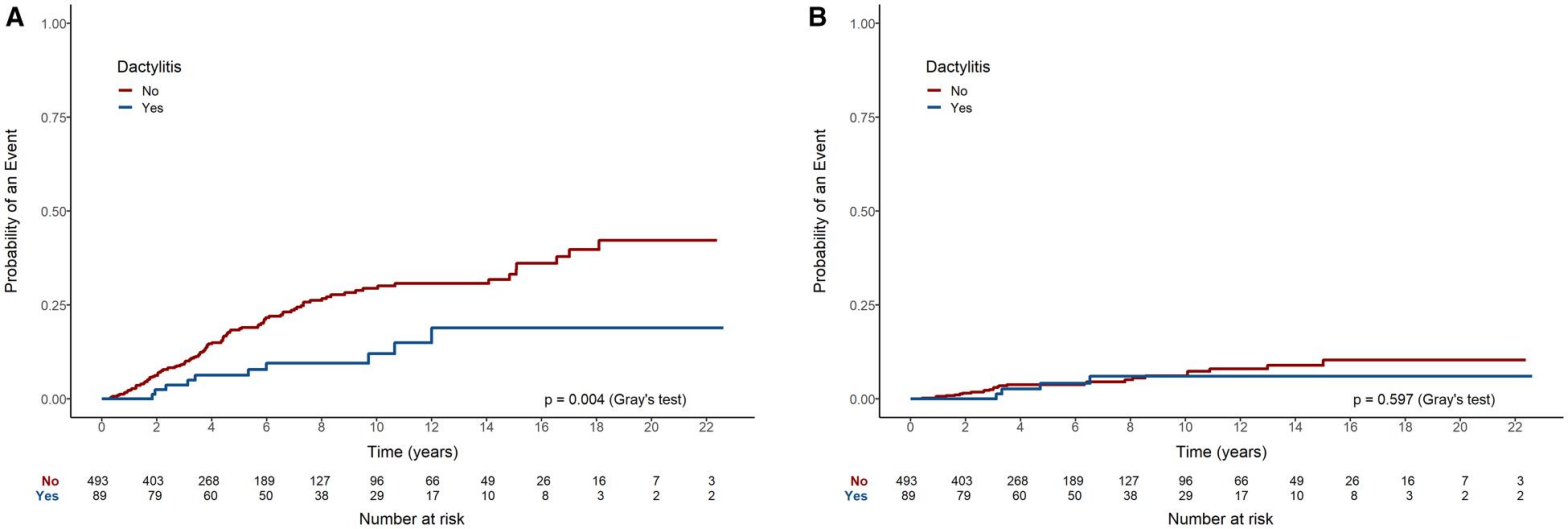
Occurrence of treatment discontinuation over time in patients with dactylitis due to **(A)** lack of effectiveness and **(B)** side effects

Patients with psoriasis presented a lower occurrence of treatment discontinuation over time due to lack of effectiveness compared with those without psoriasis (*P* < 0.001) (Fig. 2A). After 5 years, 8% of patients with psoriasis were inferred to have stopped their biologic medication due to lack of effectiveness, compared with 19% for those without psoriasis; by 10 years, this increased to 9% for patients with psoriasis and 31% for those without. The occurrence of treatment discontinuation due to side effects was similar between patients with and without psoriasis (*P* = 0.465) (Fig. 2B). The cumulative incidence at 5 years was inferred to be 2% and 4%, respectively. Comparable results were noted when comparing subjects with dactylitis to those without: patients with dactylitis showed a significantly lower incidence of medication discontinuation due to lack of effectiveness (*P* = 0.004) (Fig. 3A), and no significant difference between subjects with and without dactylitis was observed when treatment cessation was due to side effects (*P* = 0.597) (Fig. 3B). After 5 years, 6% of subjects with dactylitis were inferred to have discontinued their biologic medication due to lack of effectiveness, compared with 19% for those without the disease manifestation; by 10 years, this increased to 12% and 29% for patients with and without dactylitis, respectively.

For patients with and without a history of nail psoriasis, any EMM or any peripheral musculoskeletal feature, a non-significant trend toward a lower rate of treatment discontinuation due to lack of effectiveness was observed for those with the disease manifestation, but this trend was not seen for discontinuation due to side effects (Supplementary Figs S3, S4, S7, available at *Rheumatology Advances in Practice* online). A similar occurrence of treatment cessation according to reasons for discontinuation was observed between patients with and without AAU, IBD, peripheral arthritis and enthesitis, as depicted in Supplementary Figs S1, S2, S5 and S6, available at *Rheumatology Advances in Practice* online.

## Discussion

In this real-world observational cohort study of nearly 600 axSpA patients, we aimed to assess the impact of EMMs and peripheral musculoskeletal features on first biologic drug survival. We found that patients with psoriasis, nail psoriasis, any EMM, dactylitis or at least one peripheral musculoskeletal feature presented prolonged drug survival compared with those without these disease manifestations. Conversely, the presence of AAU, IBD, peripheral arthritis and enthesitis did not influence bDMARD retention. After adjusting for confounding, psoriasis and any peripheral musculoskeletal feature were independently associated with a protective effect on drug discontinuation, showing consistency between results. The observed effect on drug survival was only seen in participants who discontinued the drug due to lack of effectiveness.

Only a few previous studies have investigated the impact of EMMs and peripheral musculoskeletal features on biologic drug survival. It should be noted, however, that results have been conflicting and, to our knowledge, no previous study has included patients with non-radiographic axSpA. In a Swedish observational study of 2577 AS patients by Lindström *et al.* [15], the presence of AAU was associated with a longer biologic drug survival, while psoriasis shortened the retention of TNFi. A diagnosis of IBD did not influence biologic drug survival. Due to a higher number of patients, more variables were included in their model than was possible in our study. However, their results were not significantly altered by these adjustments. In contrast, a cohort study of 1078 subjects diagnosed with AS in Korea used a different methodology, dividing their cohort into two groups at specific time points: those who continued their TNFi and those who discontinued it [16]. They reported that psoriasis, peripheral arthritis and enthesitis were associated with discontinuation of the first TNFi in a univariate Cox regression analysis, with peripheral arthritis remaining predictive of TNFi therapy discontinuation after adjusting for other extra-articular manifestations in a multivariate Cox regression analysis.

In contrast to these previous research findings, our study revealed longer drug survival in patients with psoriasis. One explanation for this is that our cohort may have unwittingly included a proportion of patients with axial PsA. Past studies have shown a better TNFi retention rate in PsA compared with AS [29, 30] and therefore we hypothesize that this may explain the longer biologic drug survival observed in our study. It should be noted that 19% of our cohort had concomitant psoriasis, which is higher than the prevalence in the aforementioned Swedish and Korean studies (6% and 2.7%, respectively). The prevalence of psoriasis in our cohort also exceeds the 9.3% reported in a large metanalysis of patients with axSpA [31]. Up to 6% of our patients also presented with nail psoriasis. Additionally, patients with psoriasis and nail psoriasis in our study presented a high prevalence of dactylitis (22% and 30%, respectively), a clinical feature more commonly associated with PsA with axial involvement than axSpA with or without psoriasis [32, 33].

Our study included patients on either a TNFi or IL-17i prescribed as a first bDMARD. IL-17i is known to be more effective in treating psoriasis than TNFi and it could be hypothesized that it may positively influence drug survival [34], however, a recent retrospective study of 147 AS patients on secukinumab did not find an association between psoriasis and drug survival [18]. In our study, psoriasis remained significantly associated with a lower risk for drug discontinuation after adjusting for the class of bDMARD. Given that the availability of IL-17i is more recent, fewer patients were on IL-17i in our study, thus limiting the comparison with subjects on TNFi. Further data will be required to assess class effect on drug survival.

We did not observe an association between the presence of AAU and bDMARD retention, consistent with previous observations [16]. Additionally, previous studies investigating predictors of TNFi treatment response did not identify uveitis as a significant factor, which further supports our findings [9, 10]. The lack of effect of IBD on biologic drug survival also aligns with conclusions from other studies [15, 16].

Our study showed that the presence of at least one peripheral musculoskeletal feature was significantly associated with a longer biologic drug survival and associated with a lower risk of discontinuation even after adjusting for confounding variables. When looking at the individual peripheral musculoskeletal features, namely peripheral arthritis, enthesitis and dactylitis, only dactylitis was associated with longer bDMARD retention in the univariable analysis. Previous data from the Korean College of Rheumatology Biologics registry showed a shorter TNFi survival in AS patients with peripheral arthritis and enthesitis, but not for those with dactylitis [16]. Another study used a cluster analysis to classify 1042 axSpA patients on TNFi in two groups: isolated axial *vs* extra-axial manifestations [7]. The extra-axial group had a significantly lower drug survival than the axial group. In contrast, a cohort study of 242 AS patients on TNFi in Sweden found that the presence of peripheral arthritis was a significant predictor for better drug survival [35]. Regional differences in clinical phenotypes of axSpA [36] and national prescribing habits could explain these differing results. Further studies are needed to clarify how peripheral musculoskeletal features impact biologic drug survival.

There may be other potential explanations for our findings. First, the positive effect of a particular bDMARD on a patient’s disease manifestation (such as psoriasis) may lead them to be reluctant to change medication, even if the axial disease is uncontrolled. Second, these manifestations could be associated with higher disease activity, which has been associated with better treatment response [37]. Third, the presence of an EMM or a peripheral musculoskeletal manifestation may decrease the likelihood of misdiagnosing a non-inflammatory musculoskeletal disorder as axSpA, thereby improving the probability of response to bDMARDs.

Using a competing risk analysis, we found that patients with psoriasis or dactylitis presented a lower rate of treatment discontinuation due to a lack of effectiveness compared with those without the corresponding disease manifestations, but the occurrence of treatment discontinuation due to side effects was similar. Although not statistically significant, a similar trend was observed for patients with nail psoriasis, any EMM or any peripheral musculoskeletal feature. Therefore, our results may suggest that the prolonged biologic drug survival for these disease manifestations could be attributed to enhanced effectiveness of treatment rather than reduced side effects.

This study is not without limitation. First, diagnoses of axSpA were not objectively confirmed with central-read imaging or other standardized techniques. However, all investigators were rheumatologists with extensive experience in the care of axSpA, therefore misclassification would be uncommon, but would be representative of real-world experience. Second, disease manifestations were recorded by rheumatologists, which could also have introduced a risk of misclassification. This may be especially true for EMMs since these manifestations are typically diagnosed by other specialists. Thus, non-differential misclassification could have occurred, which would result in an underestimation of the observed effect. Third, the higher prevalence of psoriasis in our axSpA cohort compared with what is typically observed may limit the generalizability of our study findings. Fourth, the impact of disease manifestations on drug survival was assessed as an exposed or never-exposed variable and the impact of disease manifestation over time could not be assessed. Fifth, we could not include all potential predictors of prolonged biologic survival in our multivariable analysis, such as baseline disease activity, CRP or additional comorbidities [15]. Sixth, LOCF is a widely used method for imputing missing follow-up data in longitudinal studies, assuming the last observed value remains unchanged until discontinuation occurs in our data; however, this assumption can introduce bias. To assess the robustness of our findings, we performed a sensitivity analysis using other imputation methods (data not shown). In the sensitivity analysis, the results of the multivariable Cox model were generally consistent with those obtained from the LOCF method, suggesting that our findings are not highly sensitive to the choice of imputation method. Finally, there was no assessment for patient adherence to treatment in our database, which is a key component in assessing biologic drug survival. Nevertheless, non-adherence is prevalent in the real world and its inclusion in our study enhances the generalizability of our findings. Overall, these limitations were counterbalanced by the strengths of this study, which included a large cohort of well-characterized axSpA patients, both radiographic and non-radiographic, and rigorous assessment of drug survival to first TNFi or IL-17i according to various disease manifestations.

In conclusion, our study showed that various axSpA disease manifestations had an impact on first biologic drug survival. Specifically, we found that psoriasis and the presence of at least one peripheral musculoskeletal feature were independently associated with a reduced risk for discontinuation. For psoriasis, this effect could be driven by greater efficacy rather than tolerability, as subjects with psoriasis presented a lower occurrence of treatment discontinuation due to inefficacy, but not side effects. Future research will be needed to further refine treatment strategies in axSpA according to specific disease manifestations.

## Supplementary Material

rkaf004_Supplementary_Data

## Data Availability

The study dataset is not publicly available, but it is available from the corresponding author upon reasonable request.
