# Supplementary material for: Impact of disease manifestations on first biologic drug survival in axial spondyloarthritis: a real-life Canadian study
Source: Rheumatol Adv Pract. 2025 Jan 8;9(1):rkaf004. doi: 10.1093/rap/rkaf004 (PMC11780884; doi:10.1093/rap/rkaf004)
Supplement: rkaf004_Supplementary_Data [file rkaf004_supplementary_data.zip › 00c0b_24-176 Supplementary Material.docx]

Supplementary Material

Supplementary Table S1**:** Demographic and disease characteristics at biologic initiation according to the presence of AAU, IBD or psoriasis

| Variables | AAU^a^ | | | | IBD^a^ | | | | Psoriasis^a^ | | | | |
| --- | --- | --- | --- | --- | --- | --- | --- | --- | --- | --- | --- | --- | --- |
|  | Present  (n=196)^b^ | Missing Data | Absent  (n=396) ^b^ | Missing Data | Present  (n=101) ^b^ | Missing  Data | Absent  (n=492) ^b^ | Missing Data | Present  (n=110) ^b^ | Missing  Data | Absent  (n=482) ^b^ | Missing Data | |
| Age, mean (SD), years | 42.0 (13.4) | 2 | 36.0 (12.8) | 9 | 39.5 (13.2) | 3 | 37.7 (13.3) | 8 | 39.9 (13.5) | 1 | 37.5 (13.3) | 10 | |
| Male sex, n (%) | 128 (66) | 1 | 248 (64) | 7 | 66 (66) | 1 | 326 (64) | 7 | 75 (69) | 1 | 301 (63) | 7 | |
| White, n (%) | 157 (81) | 2 | 313 (82) | 13 | 90 (91) | 2 | 380 (79) | 13 | 93 (85) | 0 | 377 (81) | 15 | |
| Smoking ever, n (%) | 71 (37) | 4 | 166 (43) | 7 | 50 (50) | 0 | 188 (39) | 11 | 51 (47) | 1 | 186 (39) | 10 | |
| Body Mass Index, mean (SD) | 27.4 (5.6) | 95 | 27.6 (7.2) | 169 | 25.5 (5.6) | 43 | 28.0 (6.9) | 222 | 27.0 (5.4) | 57 | 27.6 (7.0) | 207 | |
| Anxiety and/or  depression, n (%) | 82 (42) | 1 | 152 (39) | 6 | 44 (45) | 4 | 190 (39) | 4 | 42 (38) | 0 | 192 (40) | 7 | |
| Disease duration, mean (SD), years | 18.4 (11.9) | 13 | 13.2 (10.0) | 38 | 15.7 (11.9) | 10 | 14.8 (10.8) | 41 | 15.7 (11.2) | 5 | 14.8 (10.9) | 46 | |
| HLA-B27 positive, n (%) | 142 (85) | 28 | 248 (72) | 52 | 58 (68) | 16 | 332 (78) | 65 | 77 (79) | 13 | 313 (75) | 67 | |
| Radiographic axSpA, n (%) | 170 (88) | 3 | 330 (86) | 14 | 86 (87) | 2 | 415 (87) | 15 | 99 (93) | 4 | 401 (86) | 13 | |
| CRP, mean (SD), mg/L | 14.8 (17.4) | 95 | 13.9 (20.6) | 211 | 13.0 (17.8) | 55 | 14.4 (19.9) | 252 | 16.0 (22.1) | 55 | 13.8 (18.9) | 251 | |
| BASDAI, mean (SD) | 5.6 (2.2) | 92 | 5.2 (2.2) | 215 | 5.1 (2.5) | 55 | 5.3 (2.2) | 252 | 5.1 (2.5) | 61 | 5.4 (2.2) | 246 | |
| ASDAS-CRP, mean (SD) | 3.5 (1.1) | 125 | 3.2 (1.1) | 279 | 3.4 (1.2) | 71 | 3.3 (1.1) | 334 | 3.3 (1.0) | 79 | 3.3 (1.1) | 325 | |
| BASFI, mean (SD) | 4.1 (2.7) | 94 | 4.2 (2.5) | 215 | 3.9 (2.6) | 54 | 4.2 (2.6) | 256 | 4.1 (2.7) | 59 | 4.2 (2.6) | 250 | |
| BASMI, mean (SD) | 3.0 (1.8) | 86 | 2.9 (1.5) | 210 | 3.2 (1.6) | 52 | 2.9 (1.6) | 244 | 3.1 (1.4) | 55 | 2.9 (1.6) | 241 | |
| ASQoL, mean (SD) | 8.5 (5.5) | 98 | 9.2 (5.4) | 234 | 8.3 (5.7) | 59 | 9.1 (5.4) | 274 | 8.3 (5.6) | 65 | 9.1 (5.4) | 267 | |
| SF-36PCS, mean (SD) | 35.2 (10.1) | 109 | 34.7 (10.3) | 242 | 33.0 (10.4) | 61 | 35.2 (10.2) | 291 | 34.8 (10.2) | 63 | 34.9 (10.3) | 288 | |
| SF-36MCS, mean (SD) | 44.6 (11.6) | 109 | 44.7 (11.9) | 242 | 46.7 (12.1) | 61 | 44.3 (11.7) | 291 | 45.2 (12.1) | 63 | 44.5 (11.7) | 288 | |
| Infliximab, n (%) | 34 (17) | 0 | 94 (24) |  | 38 (38) | 0 | 90 (18) | 0 | 37 (34) | 0 | 91 (19) | 0 | |
| Etanercept, n (%) | 41 (21) | 0 | 71 (18) |  | 7 (7) | 0 | 105 (21) | 0 | 21 (19) | 0 | 91 (19) | 0 | |
| Adalimumab, n (%) | 88 (45) | 0 | 132 (33) |  | 47 (47) | 0 | 173 (35) | 0 | 34 (31) | 0 | 186 (39) | 0 | |
| Golimumab, n (%) | 29 (15) | 0 | 68 (17) |  | 7 (7) | 0 | 90 (18) | 0 | 14 (13) | 0 | 83 (17) | 0 | |
| Certolizumab, n (%) | 0 (0) | 0 | 13 (3) |  | 1 (1) | 0 | 12 (2) | 0 | 1 (1) | 0 | 12 (2) | 0 | |
| Ixekizumab, n (%) | 1 (0) | 0 | 0 (0) |  | 0 (0) | 0 | 1 (0) | 0 | 0 (0) | 0 | 1 (0) | 0 | |
| Secukinumab, n (%) | 3 (2) | 0 | 18 (5) |  | 1 (1) | 0 | 21 (4) | 0 | 3 (3) | 0 | 18 (4) | 0 | |
| csDMARD, n (%) | 37 (19) | 0 | 54 (14) | 0 | 16 (16) | 0 | 75 (15) | 0 | 18 (16) | 0 | 73 (15) | 0 | |
| AAU ever, n (%) | 196 (100) | 0 | 0 (0) | 0 | 36 (36) | 0 | 160 (33) | 1 | 41 (37) | 0 | 155 (32) | 0 | |
| IBD ever, n (%) | 36 (18) | 0 | 65 (16) | 0 | 101 (100) | 0 | 0 (0) |  | 30 (27) | 0 | 71 (15) | 0 | |
| Psoriasis ever, n (%) | 41 (21) | 0 | 69 (17) | 0 | 30 (30) | 0 | 80 (16) | 1 | 110(100) | 0 | 0 (0) | 0 | |
| Nail psoriasis ever, n (%) | 14 (7) | 0 | 19 (5) | 0 | 8 (8) | 0 | 25 (5) | 1 | 22 (20) | 0 | 11 (2) | 0 | |
| Any EMM ever, n (%) | 196 (100) | 0 | 126 (32) | 0 | 101 (100) | 0 | 221 (45) | 0 | 110 (100) | 0 | 212 (44) | 0 | |
| Peripheral arthritis ever, n (%) | 31 (16) | 6 | 66 (18) | 23 | 23 (24) | 5 | 74 (16) | 25 | 15 (14) | 2 | 82 (18) | 27 | |
| Enthesitis ever, n (%) | 107 (55) | 0 | 174 (44) | 0 | 45 (45) | 0 | 236 (48) | 0 | 54 (49) | 0 | 227 (47) | 0 | |
| Dactylitis ever, n (%) | 35 (18) | 0 | 56 (14) | 0 | 14 (14) | 0 | 77 (16) | 0 | 24 (22) | 0 | 67 (14) | 0 | |
| Any peripheral musculoskeletal feature ever, n (%) | 141 (72) | 0 | 279 (70) | 0 | 72 (71) | 0 | 348 (71) | 0 | 86 (78) | 0 | 334 (69) | 0 | |
| ^a^Continuous variables presented as mean (standard deviation) and categorical variables as count.  ^b^Inconsistencies in sample size are due to missing values.  ^b^Age, sex, race, body mass index, disease duration, HLAB27, radiographic classification, CRP, BASDAI, ASDAS-CRP,BASFI, BASMI, ASQoL, SF-36PCS, SF-36MCS, and medication data were baseline data; while smoking, anxiety and/or depression, and disease manifestations were exposed/never exposed variables.  SD, standard deviation; HLA-B27, Human Leukocyte Antigen B27; axSpA, axial spondyloarthritis; CRP, C-reactive protein; BASDAI, Bath Ankylosing Spondylitis Disease Activity Index; ASDAS-CRP, Ankylosing Spondylitis Disease Activity Score-CRP; BASFI, Bath Ankylosing Spondylitis Functional Index; BASMI, Bath Ankylosing Spondylitis Metrology Index; ASQoL, Ankylosing Spondylitis Quality of Life questionnaire; SF-36PCS, Short-Form Health Survey physical component; SF-36MCS, Short-Form Health Survey physical mental component; csDMARD, Conventional Synthetic Disease-Modifying Antirheumatic Drug; AAU, Acute Anterior Uveitis; IBD, Inflammatory Bowel Disease; EMM, Extra-Musculoskeletal Manifestation. | | | | | | | | | | | | |  |

Supplementary Table S2**:** Demographic and disease characteristics at biologic initiation according to the presence of nail psoriasis, any EMM or peripheral arthritis

| Variables | Nail Psoriasis^a^ | | | | Any EMM^a^ | | | | Peripheral Arthritis^a^ | | | | |
| --- | --- | --- | --- | --- | --- | --- | --- | --- | --- | --- | --- | --- | --- |
|  | Present  (n=33) ^b^ | Missing Data | Absent  (n=559) ^b^ | Missing Data | Present  (n=322) ^b^ | Missing  Data | Absent  (n=271) ^b^ | Missing Data | Present  (n=97) ^b^ | Missing  Data | Absent  (n=466) ^b^ | Missing Data | |
| Age, mean (SD), years | 41.6 (13.1) | 0 | 37.8 (13.3) | 11 | 40.3 (13.4) | 5 | 35.1 (12.7) | 6 | 39.1 (13.1) | 1 | 37.7 (13.4) | 8 | |
| Male sex, n (%) | 22 (67) | 0 | 354 (64) | 8 | 211 (66) | 3 | 166 (62) | 5 | 56 (59) | 2 | 307 (66) | 4 | |
| White, n (%) | 27 (82) | 0 | 443 (81) | 15 | 261 (82) | 4 | 209 (80) | 11 | 75 (77) | 0 | 370 (82) | 14 | |
| Smoking ever, n (%) | 16 (50) | 1 | 221 (40) | 10 | 138 (44) | 5 | 100 (38) | 6 | 42 (44) | 1 | 181 (40) | 9 | |
| Body Mass Index, mean (SD) | 26.7 (5.0) | 16 | 27.6 (6.8) | 248 | 27.0 (5.8) | 149 | 28.1 (7.7) | 116 | 27.9 (6.5) | 33 | 27.3 (6.6) | 226 | |
| Anxiety and/or  depression, n (%) | 14 (42) | 0 | 220 (40) | 7 | 128 (40) | 4 | 106 (40) | 4 | 58 (60) | 0 | 168 (36) | 1 | |
| Disease duration, mean (SD), years | 15.4 (12.3) | 1 | 14.9 (10.9) | 50 | 16.5 (11.8) | 25 | 13.1 (9.6) | 26 | 14.5 (12.0) | 14 | 15.0 (10.7) | 35 | |
| HLA-B27 positive, n (%) | 22 (79) | 5 | 368 (76) | 75 | 216 (78) | 46 | 174 (74) | 35 | 53 (67) | 18 | 323 (79) | 56 | |
| Radiographic axSpA, n (%) | 30 (94) | 1 | 470 (87) | 16 | 277 (88) | 8 | 224 (85) | 9 | 78 (86) | 6 | 401 (88) | 10 | |
| CRP, mean (SD), mg/L | 17.2 (20.5) | 20 | 14.1 (19.5) | 286 | 15.7 (20.3) | 168 | 12.5 (18.6) | 139 | 14.9 (19.0) | 58 | 14.5 (20.1) | 233 | |
| BASDAI, mean (SD) | 5.0 (2.6) | 19 | 5.3 (2.2) | 288 | 5.3 (2.3) | 169 | 5.3 (2.1) | 138 | 5.3 (2.3) | 58 | 5.3 (2.2) | 237 | |
| ASDAS-CRP, mean (SD) | 3.5 (1.3) | 25 | 3.3 (1.1) | 379 | 3.4 (1.1) | 216 | 3.2 (1.1) | 189 | 3.1 (1.0) | 70 | 3.4 (1.1) | 314 | |
| BASFI, mean (SD) | 3.7 (3.2) | 18 | 4.2 (2.6) | 291 | 4.0 (2.7) | 170 | 4.3 (2.5) | 140 | 4.6 (2.4) | 60 | 4.0 (2.6) | 236 | |
| BASMI, mean (SD) | 2.8 (1.4) | 18 | 3.0 (1.6) | 278 | 3.1 (1.7) | 158 | 2.9 (1.5) | 138 | 2.9 (1.9) | 63 | 3.0 (1.5) | 221 | |
| ASQoL, mean (SD) | 7.5 (5.4) | 20 | 9.0 (5.5) | 312 | 8.4 (5.4) | 180 | 9.7 (5.4) | 153 | 10.1 (5.8) | 62 | 8.7 (5.3) | 258 | |
| SF-36PCS, mean (SD) | 35.7 (11.2) | 21 | 34.8 (10.2) | 330 | 34.4 (10.5) | 192 | 35.4 (10.0) | 160 | 34.3 (10.0) | 64 | 35.1 (10.4) | 271 | |
| SF-36MCS, mean (SD) | 47.8 (12.4) | 21 | 44.5 (11.7) | 330 | 45.6 (11.7) | 192 | 43.6 (11.8) | 160 | 43.1 (13.2) | 64 | 45.1 (11.3) | 271 | |
| Infliximab, n (%) | 11 (33) | 0 | 117 (21) | 0 | 79 (25) | 0 | 49 (18) | 0 | 17 (18) | 0 | 105 (23) | 0 | |
| Etanercept, n (%) | 8 (24) | 0 | 104 (19) | 0 | 60 (19) | 0 | 52 (19) | 0 | 8 (8) | 0 | 95 (20) | 0 | |
| Adalimumab, n (%) | 11 (33) | 0 | 209 (37) | 0 | 136 (42) | 0 | 84 (31) | 0 | 50 (52) | 0 | 160 (34) | 0 | |
| Golimumab, n (%) | 1 (3) | 0 | 96 (17) | 0 | 40 (12) | 0 | 57 (21) | 0 | 17 (18) | 0 | 78 (17) | 0 | |
| Certolizumab, n (%) | 0 (0) | 0 | 13 (2) | 0 | 2 (1) | 0 | 11 (4) | 0 | 2 (2) | 0 | 9 (2) | 0 | |
| Ixekizumab, n (%) | 0 (0) | 0 | 1 (0) | 0 | 1 (0) | 0 | 0 (0) | 0 | 0 (0) | 0 | 1 (0) | 0 | |
| Secukinumab, n (%) | 2 (6) | 0 | 19 (3) | 0 | 4 (1) | 0 | 18 (7) | 0 | 3 (3) | 0 | 18 (4) | 0 | |
| csDMARD, n (%) | 7 (21) | 0 | 84 (15) | 0 | 56 (17) | 0 | 35 (13) | 0 | 19 (20) |  | 67 (14) | 0 | |
| AAU ever, n (%) | 14 (42) | 0 | 182 (33) | 0 | 196 (61) | 0 | 0 (0) | 1 | 31 (32) | 0 | 159 (34) | 0 | |
| IBD ever, n (%) | 8 (24) | 0 | 93 (17) | 0 | 101 (31) | 0 | 0 (0) | 0 | 23 (24) | 0 | 73 (16) | 0 | |
| Psoriasis ever, n (%) | 22 (67) | 0 | 88 (16) | 0 | 196 (61) | 0 | 0 (0) | 1 | 15 (15) | 0 | 93 (20) | 0 | |
| Nail psoriasis ever, n (%) | 33 (100) | 0 | 0 (0) | 0 | 33 (10) | 0 | 0 (0) | 1 | 3 (3) | 0 | 29 (6) | 0 | |
| Any EMM ever, n (%) | 33 (100) | 0 | 289 (52) | 0 | 322 (100) | 0 | 0 (0) | 0 | 54 (56) | 0 | 258 (55) | 0 | |
| Peripheral arthritis ever, n (%) | 3 (9) | 1 | 94 (18) | 28 | 54 (17) | 10 | 43 (17) | 20 | 281 (100) | 0 | 0 (0) | 0 | |
| Enthesitis ever, n (%) | 22 (67) | 0 | 259 (46) | 0 | 161 (50) | 0 | 120 (44) | 0 | 55 (57) | 0 | 212 (45) | 0 | |
| Dactylitis ever, n (%) | 10 (30) | 0 | 81 (14) | 0 | 53 (16) | 0 | 38 (14) | 0 | 25 (26) | 0 | 60 (13) | 0 | |
| Any peripheral musculoskeletal feature ever, n (%) | 30 (91) | 0 | 390 (70) | 0 | 234 (73) | 0 | 186 (69) | 0 | 97 (100) | 0 | 300 (64) | 0 | |
| ^a^Continuous variables presented as mean (standard deviation) and categorical variables as count.  ^b^Inconsistencies in sample size are due to missing values.  ^b^Age, sex, race, body mass index, disease duration, HLAB27, radiographic classification, CRP, BASDAI, ASDAS-CRP,BASFI, BASMI, ASQoL, SF-36PCS, SF-36MCS, and medication data were taken at baseline data; while smoking, anxiety and/or depression, and disease manifestations were exposed/never exposed variables.  SD, standard deviation; HLA-B27, Human Leukocyte Antigen B27; axSpA, axial spondyloarthritis; CRP, C-reactive protein; BASDAI, Bath Ankylosing Spondylitis Disease Activity Index; ASDAS-CRP, Ankylosing Spondylitis Disease Activity Score-CRP; BASFI, Bath Ankylosing Spondylitis Functional Index; BASMI, Bath Ankylosing Spondylitis Metrology Index; ASQoL, Ankylosing Spondylitis Quality of Life questionnaire; SF-36PCS, Short-Form Health Survey physical component; SF-36MCS, Short-Form Health Survey physical mental component; csDMARD, Conventional Synthetic Disease-Modifying Antirheumatic Drug; AAU, Acute Anterior Uveitis; IBD, Inflammatory Bowel Disease; EMM, Extra-Musculoskeletal Manifestation. | | | | | | | | | | | | |  |

Supplementary Table S3**:** Demographic and disease characteristics at biologic initiation according to the presence of enthesitis, dactylitis or any peripheral feature

| Variables | Enthesitis^a^ | | | | Dactylitis^a^ | | | | Any Peripheral Musculoskeletal Feature^a^ | | | | |
| --- | --- | --- | --- | --- | --- | --- | --- | --- | --- | --- | --- | --- | --- |
|  | Present  (n=281) ^b^ | Missing Data | Absent  (n=312) ^b^ | Missing Data | Present  (n=91) ^b^ | Missing  Data | Absent  (n=502) ^b^ | Missing Data | Present  (n=420) ^b^ | Missing  Data | Absent  (n=173) ^b^ | Missing Data | |
| Age, mean (SD), years | 39.6 (13.0) | 7 | 36.5 (13.4) | 4 | 41.0 (13.8) | 2 | 37.4 (13.2) | 9 | 38.8 (13.2) | 9 | 35.9 (13.5) | 2 | |
| Male sex, n (%) | 156 (57) | 6 | 221 (71) | 2 | 54 (61) | 2 | 323 (65) | 6 | 253 (61) | 8 | 124 (72) | 0 | |
| White, n (%) | 229 (83) | 6 | 241 (80) | 9 | 73 (81) | 1 | 397 (81) | 14 | 341 (83) | 7 | 129 (78) | 8 | |
| Smoking ever, n (%) | 104 (38) | 5 | 134 (44) | 6 | 33 (38) | 3 | 205 (41) | 0 | 169 (41) | 9 | 69 (40) | 2 | |
| Body Mass Index, mean (SD) | 27.9 (7.5) | 121 | 27.2 (6.0) | 144 | 28.4 (7.8) | 32 | 27.3 (6.5) | 233 | 27.8 (7.2) | 185 | 26.9 (5.3) | 80 | |
| Anxiety and/or  depression, n (%) | 127 (45) | 1 | 107 (35) | 7 | 46 (51) | 0 | 188 (38) | 8 | 177 (43) | 4 | 57 (34) | 4 | |
| Disease duration, mean (SD), years | 16.4 (11.2) | 26 | 13.7 (10.6) | 26 | 16.5 (12.2) | 8 | 14.7 (10.7) | 43 | 15.6 (11.2) | 39 | 13.4 (10.2) | 12 | |
| HLA-B27 positive, n (%) | 191 (82) | 48 | 199 (71) | 33 | 57 (83) | 22 | 333 (75) | 59 | 273 (77) | 66 | 117 (74) | 15 | |
| Radiographic axSpA, n (%) | 243 (89) | 8 | 258 (85) | 9 | 81 (91) | 2 | 420 (86) | 15 | 362 (89) | 13 | 139 (82) | 4 | |
| CRP, mean (SD), mg/L | 14.1 (18.8) | 128 | 14.3 (20.4) | 179 | 16.2 (22.3) | 43 | 13.8 (19.0) | 264 | 15.1 (20.6) | 208 | 11.7 (16.0) | 99 | |
| BASDAI, mean (SD) | 5.6 (2.2) | 132 | 5.0 (2.3) | 175 | 5.3 (2.4) | 46 | 5.3 (2.2) | 261 | 5.6 (2.3) | 214 | 4.7 (2.0) | 93 | |
| ASDAS-CRP, mean (SD) | 3.3 (1.1) | 181 | 3.3 (1.1) | 224 | 3.4 (1.3) | 61 | 3.3 (1.1) | 344 | 3.3 (1.1) | 279 | 3.3 (1.0) | 126 | |
| BASFI, mean (SD) | 4.2 (2.5) | 136 | 4.1 (2.7) | 174 | 3.9 (2.7) | 47 | 4.2 (2.6) | 263 | 4.4 (2.6) | 217 | 3.6 (2.6) | 93 | |
| BASMI, mean (SD) | 3.0 (1.6) | 119 | 3.0 (1.6) | 177 | 3.0 (1.7) | 41 | 3.0 (1.6) | 255 | 3.0 (1.6) | 201 | 2.8 (1.5) | 95 | |
| ASQoL, mean (SD) | 8.9 (5.5) | 148 | 9.0 (5.5) | 185 | 7.9 (6.5) | 49 | 9.2 (5.2) | 284 | 9.3 (5.6) | 233 | 8.0 (5.1) | 100 | |
| SF-36PCS, mean (SD) | 33.6 (9.8) | 156 | 36.3 (10.5) | 196 | 34.5 (10.0) | 56 | 34.9 (10.3) | 296 | 33.4 (9.8) | 242 | 39.0 (10.4) | 110 | |
| SF-36MCS, mean (SD) | 45.2 (11.7) | 156 | 44.1 (11.9) | 196 | 47.2 (13.7) | 56 | 44.2 (11.4) | 296 | 44.8 (11.9) | 242 | 44.3 (11.5) | 110 | |
| Infliximab, n (%) | 64 (23) | 0 | 64 (21) | 0 | 26 (29) | 0 | 102 (20) | 0 | 97 (23) | 0 | 31 (19) | 0 | |
| Etanercept, n (%) | 55 (20) | 0 | 57 (19) | 0 | 10 (11) | 0 | 102 (20) | 0 | 78 (19) | 0 | 34 (20) | 0 | |
| Adalimumab, n (%) | 109 (39) | 0 | 111 (36) | 0 | 41 (45) | 0 | 179 (36) | 0 | 161 (38) | 0 | 59 (34) | 0 | |
| Golimumab, n (%) | 39 (14) | 0 | 58 (19) | 0 | 10 (11) | 0 | 87 (17) | 0 | 60 (14) | 0 | 37 (21) | 0 | |
| Certolizumab, n (%) | 4 (1) | 0 | 9 (3) | 0 | 1 (1) | 0 | 12 (2) | 0 | 8 (2) | 0 | 5 (3) | 0 | |
| Ixekizumab, n (%) | 1 (0) | 0 | 0 (0) | 0 | 0 (0) | 0 | 1 (0) | 0 | 1 (0) | 0 | 0 (0) | 0 | |
| Secukinumab, n (%) | 9 (3) | 0 | 13 (4) | 0 | 3 (3) | 0 | 19 (4) | 0 | 15 (4) | 0 | 7 (4) | 0 | |
| csDMARD, n (%) | 49 (17) | 0 | 42 (13) | 0 | 24 (26) | 0 | 67 (13) | 0 | 71 (17) | 0 | 20 (12) | 0 | |
| AAU ever, n (%) | 107 (38) | 0 | 89 (29) | 1 | 35 (38) | 0 | 161 (32) | 1 | 141 (34) | 0 | 55 (32) | 1 | |
| IBD ever, n (%) | 45 (16) | 0 | 56 (18) | 0 | 14 (15) | 0 | 87 (17) | 0 | 72 (17) | 0 | 29 (17) | 0 | |
| Psoriasis ever, n (%) | 54 (19) | 0 | 56 (18) | 1 | 24 (26) | 0 | 86 (17) | 1 | 86 (20) | 0 | 24 (14) | 1 | |
| Nail psoriasis ever, n (%) | 22 (8) | 0 | 11 (4) | 1 | 10 (11) | 0 | 23 (5) | 1 | 30 (7) | 0 | 3 (2) | 1 | |
| Any EMM ever, n (%) | 161 (57) | 0 | 161 (52) | 0 | 53 (58) | 0 | 269 (54) | 0 | 234 (56) | 0 | 88 (51) | 0 | |
| Peripheral arthritis ever, n (%) | 55 (21) | 14 | 42 (14) | 16 | 25 (29) | 6 | 72 (15) | 24 | 97 (24) | 23 | 0 (0) | 0 | |
| Enthesitis ever, n (%) | 281 (100) | 0 | 0 (0) | 0 | 66 (73) | 0 | 215 (43) | 0 | 281 (67) | 0 | 0 (0) | 0 | |
| Dactylitis ever, n (%) | 66 (23) | 0 | 25 (8) | 0 | 91 (100) | 0 | 0 (0) | 0 | 91 (22) | 0 | 0 (0) | 0 | |
| Any peripheral musculoskeletal feature ever, n (%) | 281 (100) | 0 | 139 (45) | 0 | 91 (100) | 0 | 329 (66) | 0 | 420 (100) | 0 | 0 (0) | 0 | |
| ^a^Continuous variables presented as mean (standard deviation) and categorical variables as count.  ^b^Inconsistencies in sample size are due to missing values.  ^b^Age, sex, race, body mass index, disease duration, HLAB27, radiographic classification, CRP, BASDAI, ASDAS-CRP,BASFI, BASMI, ASQoL, SF-36PCS, SF-36MCS, and medication data were baseline data; while smoking, anxiety and/or depression, and disease manifestations were exposed/never exposed variables.  SD, standard deviation; HLA-B27, Human Leukocyte Antigen B27; axSpA, axial spondyloarthritis; CRP, C-reactive protein; BASDAI, Bath Ankylosing Spondylitis Disease Activity Index; ASDAS-CRP, Ankylosing Spondylitis Disease Activity Score-CRP; BASFI, Bath Ankylosing Spondylitis Functional Index; BASMI, Bath Ankylosing Spondylitis Metrology Index; ASQoL, Ankylosing Spondylitis Quality of Life questionnaire; SF-36PCS, Short-Form Health Survey physical component; SF-36MCS, Short-Form Health Survey physical mental component; csDMARD, Conventional Synthetic Disease-Modifying Antirheumatic Drug; AAU, Acute Anterior Uveitis; IBD, Inflammatory Bowel Disease; EMM, Extra-Musculoskeletal Manifestation. | | | | | | | | | | | | |  |


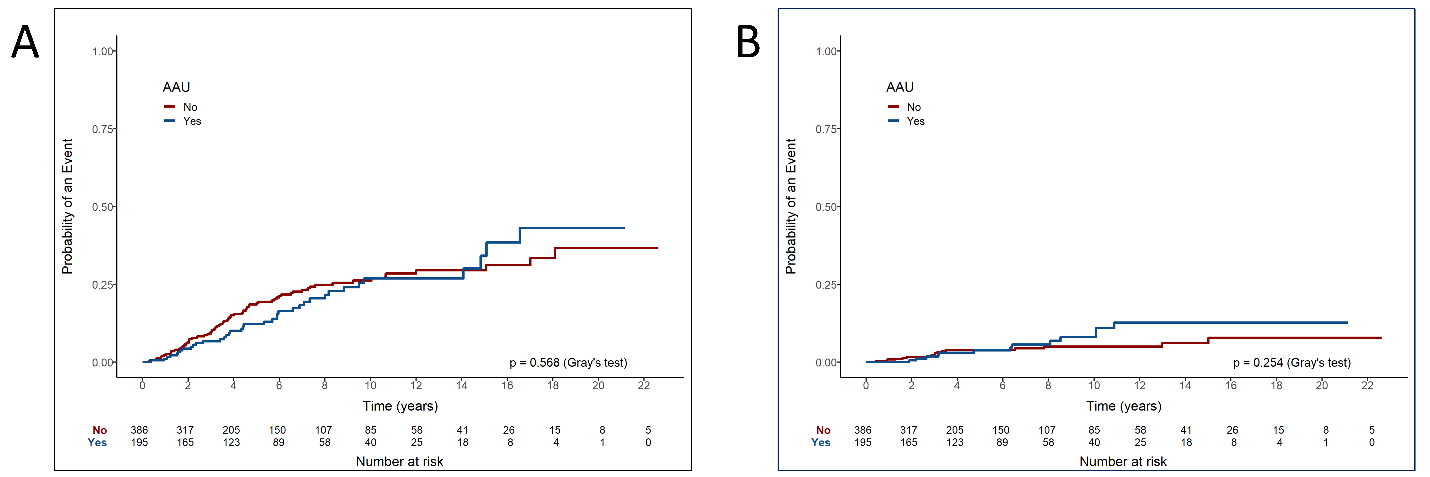


Supplementary Figure S1: Occurrence of treatment discontinuation over time in patients with acute anterior uveitis due to A) lack of effectiveness and B) side effects.


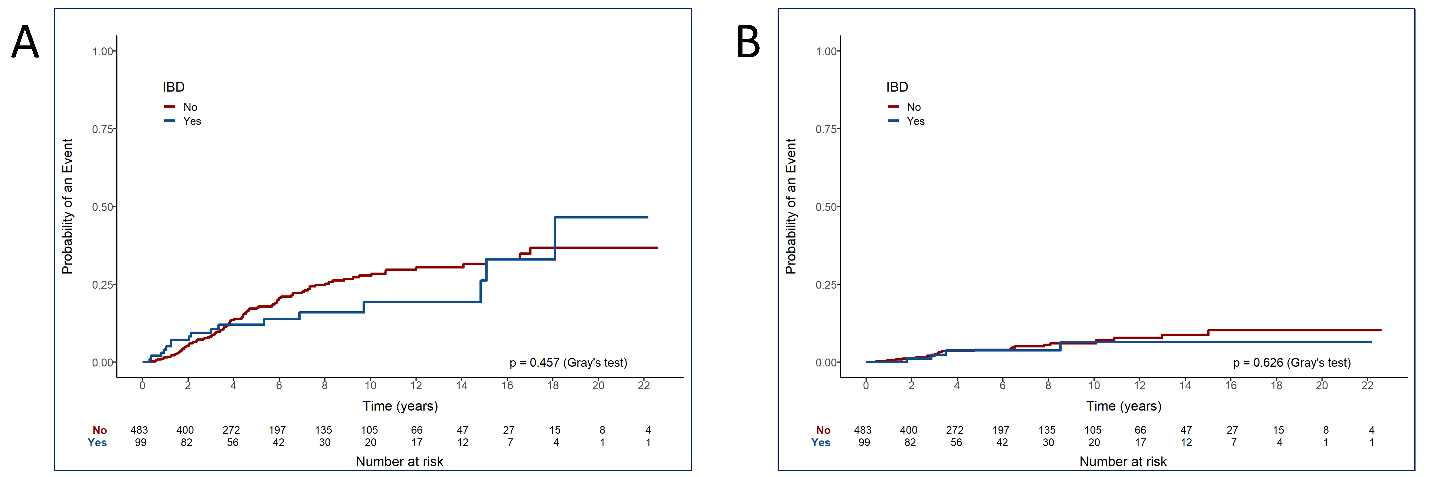


Supplementary Figure S2: Occurrence of treatment discontinuation over time in patients with inflammatory bowel disease due to A) lack of effectiveness and B) side effects.


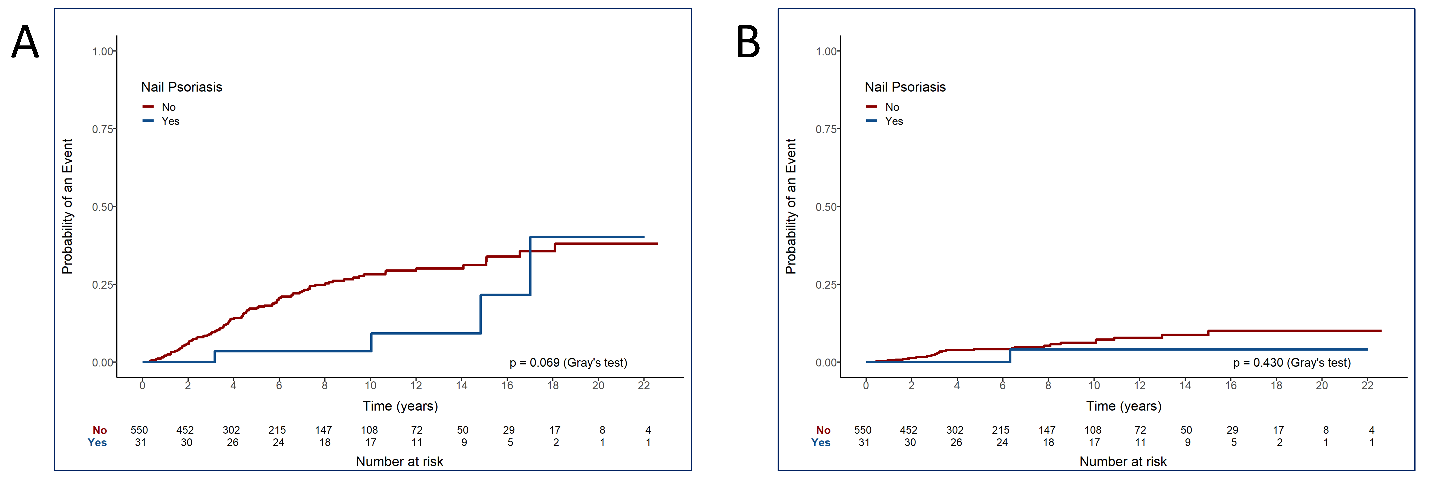


Supplementary Figure S3: Occurrence of treatment discontinuation over time in patients with nail psoriasis due to A) lack of effectiveness and B) side effects.


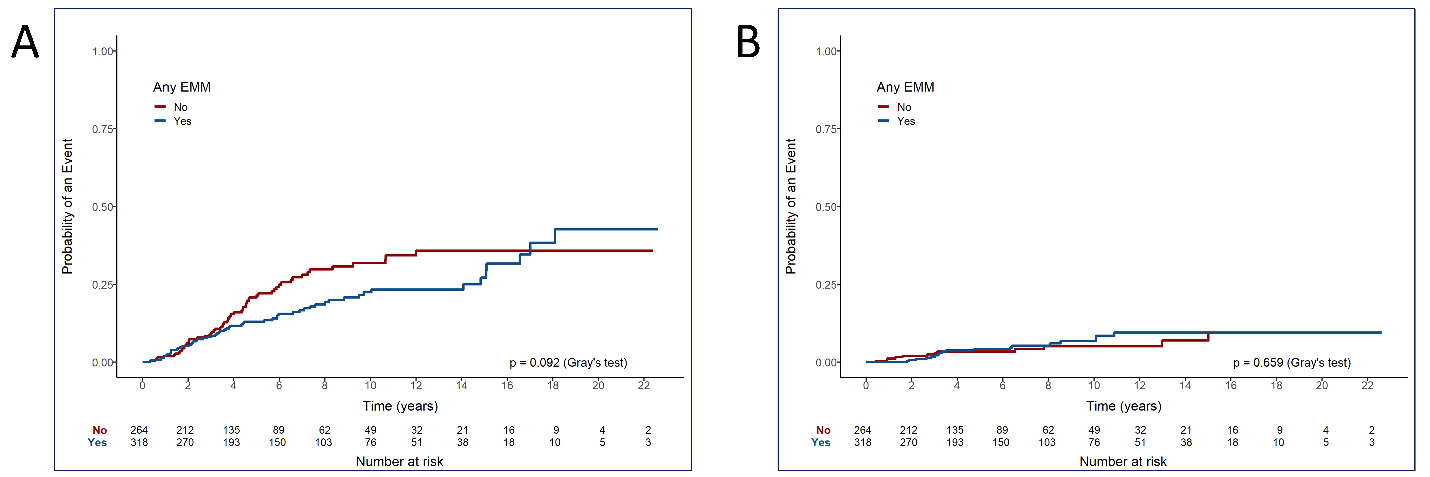


Supplementary Figure S4: Occurrence of treatment discontinuation over time in patients with any extra-musculoskeletal manifestations due to A) lack of effectiveness and B) side effects.


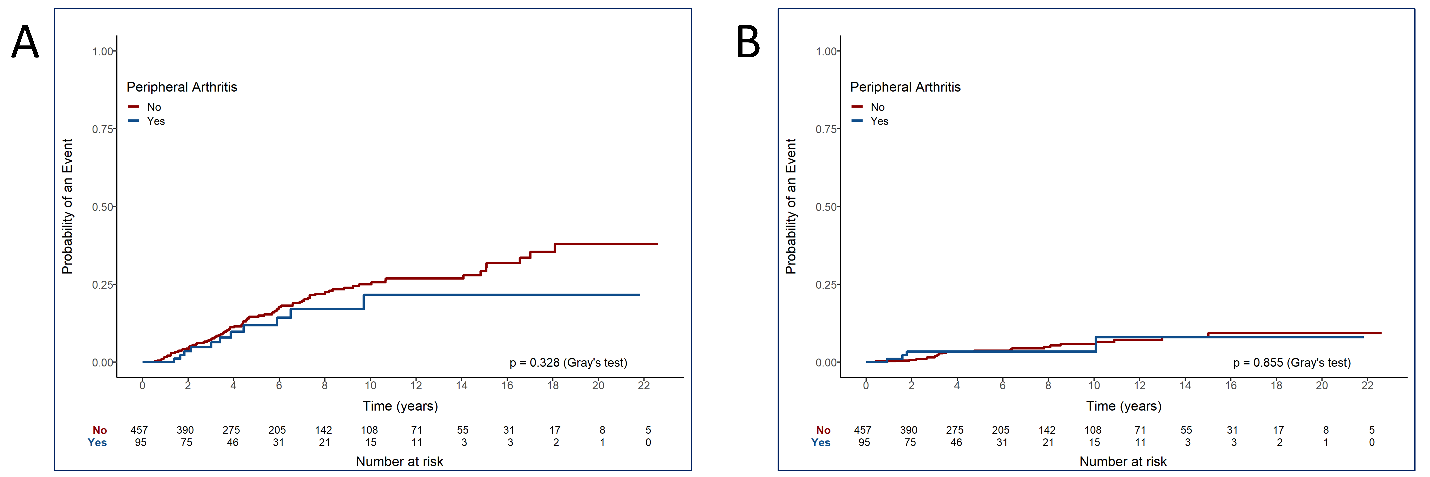


Supplementary Figure S5: Occurrence of treatment discontinuation over time in patients with peripheral arthritis due to A) lack of effectiveness and B) side effects.


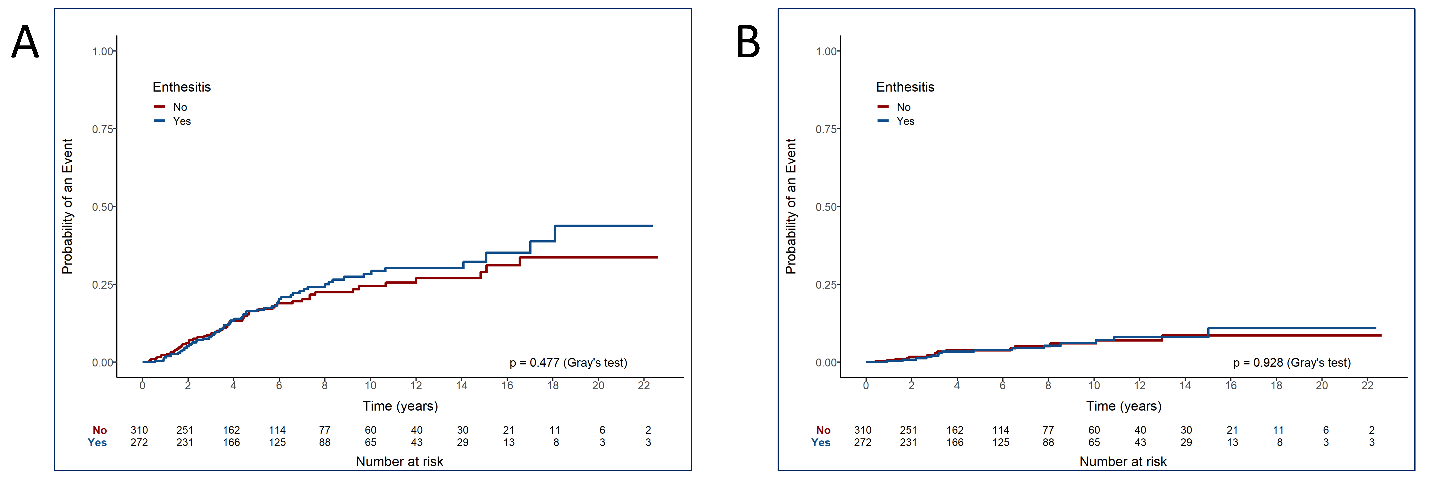


Supplementary Figure S6: Occurrence of treatment discontinuation over time in patients with enthesitis due to A) lack of effectiveness and B) side effects.


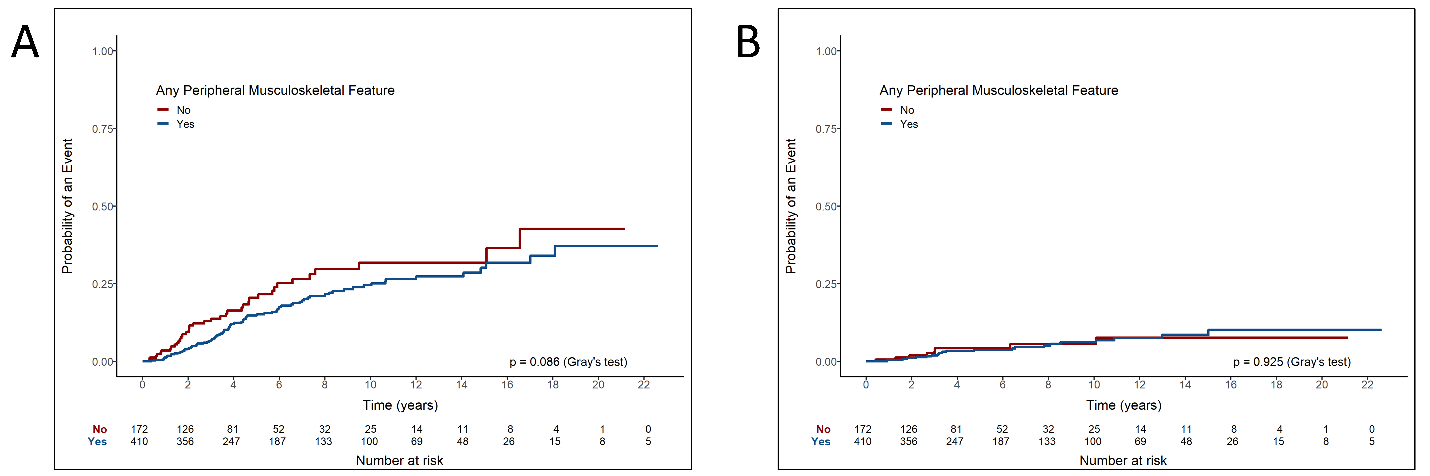


Supplementary Figure S7: Occurrence of treatment discontinuation over time in patients with any peripheral musculoskeletal feature due to A) lack of effectiveness and B) side effects.
